# Analysis of mesenchymal stem cell proteomes *in situ* in the ischemic heart

**DOI:** 10.7150/thno.47893

**Published:** 2020-09-14

**Authors:** Dunzheng Han, Junjie Yang, Eric Zhang, Yanwen Liu, Chan Boriboun, Aijun Qiao, Yang Yu, Jiacheng Sun, Shiyue Xu, Liu Yang, Wenying Yan, Bihui Luo, Dongfeng Lu, Chunxiang Zhang, Chunfa Jie, James Mobley, Jianyi Zhang, Gangjian Qin

**Affiliations:** 1 Department of Biomedical Engineering, University of Alabama at Birmingham, School of Medicine and School of Engineering, Birmingham, AL 35294, USA.; 2 Department of Cardiology, The First Affiliated Hospital of Guangzhou Medical University, Guangzhou, Guangdong 510120, China.; 3 School of Biology and Basic Medical Science, Soochow University, Suzhou, Jiangsu 215123, China.; 4 Department of Biochemistry and Nutrition, Des Moines University Medicine and Health Sciences, Des Moines, IA 50312, USA.; 5 Department of Anesthesiology and Perioperative Medicine, University of Alabama at Birmingham, School of Medicine, Birmingham, AL 35294, USA.

**Keywords:** Methionyl-tRNA synthetase, mesenchymal stem cells, myocardial infarction, proteomics, mass spectrometry

## Abstract

**Rationale:** Cell therapy for myocardial infarction is promising but largely unsuccessful in part due to a lack of mechanistic understanding. Techniques enabling identification of stem cell-specific proteomes *in situ* in the injured heart may shed light on how the administered cells respond to the injured microenvironment and exert reparative effects.

**Objective:** To identify the proteomes of the transplanted mesenchymal stem cells (MSCs) in the infarcted myocardium, we sought to target a mutant methionyl-tRNA synthetase (MetRS^L274G^) in MSCs, which charges azidonorleucine (ANL), a methionine analogue and non-canonical amino acid, to tRNA and subsequently to nascent proteins, permitting isolation of ANL-labeled MSC proteomes from ischemic hearts by ANL-alkyne based click reaction.

**Methods and Results:** Murine MSCs were transduced with lentivirus MetRS^L274G^ and supplemented with ANL; the ANL-tagged nascent proteins were visualized by bio-orthogonal non-canonical amino-acid tagging, spanning all molecular weights and by fluorescent non-canonical amino-acid tagging, displaying strong fluorescent signal. Then, the MetRS^L274G^-transduced MSCs were administered to the infarcted or Sham heart in mice receiving ANL treatment. The MSC proteomes were isolated from the left ventricular protein lysates by click reaction at days 1, 3, and 7 after cell administration, identified by LC/MS. Among all identified proteins (in Sham and MI hearts, three time-points each), 648 were shared by all 6 groups, accounting for 82±5% of total proteins in each group, and enriched under mitochondrion, extracellular exosomes, oxidation-reduction process and poly(A) RNA binding. Notably, 26, 110 and 65 proteins were significantly up-regulated and 11, 28 and 19 proteins were down-regulated in the infarcted vs. Sham heart at the three time-points, respectively; these proteins are pronounced in the GO terms of extracellular matrix organization, response to stress and regulation of apoptotic process and in the KEGG pathways of complements and coagulation cascades, apoptosis, and regulators of actin cytoskeleton.

**Conclusions:** MetRS^L274G^ expression allows successful identification of MSC-specific nascent proteins in the infarcted hearts, which reflect the functional states, adaptive response, and reparative effects of MSCs that may be leveraged to improve cardiac repair.

## Introduction

Ischemic heart disease and consequent heart failure remains the leading cause of mortality worldwide. Regenerative medicine approaches, such as cell therapy, offer exciting promise. Adult mesenchymal stem cells (MSCs) are a particularly attractive option, owing to their easy isolation and low immunogenicity and potential translational value 1, 2. However, clinical trials thus far have only shown modest benefit and a major hurdle is our limited understanding about the mechanisms underlying their biological effects 3.

Upon myocardial infarction (MI), cardiac repair initiates with an early phase of acute inflammation (~3-4 d in mice) characterized by immune cell infiltration to clear damaged cells and extracellular matrix tissue, followed by a reparative phase with resolution of inflammation, scar formation and neovascularization over the next several days 4. The biophysiological cues expressed in the injured cardiac tissue are complex, while chemokines and adhesion molecules can increase stem cell retention, excessive pro-inflammatory mediators and oxidative stress agents induce cell death, uncontrolled fibrosis and excessive extracellular matrix to contribute to the adverse cardiac remodeling and ultimately, heart failure 4. The majority of preclinical cell therapy studies chose early time points (days 1, 3 and 7 in mice) after MI, which demarcate ischemic necrosis, pro-inflammatory response, and transition to resolution of inflammation and tissue repair, respectively, and documented the differential reparative responses from MSCs 5.

MSCs are believed to enhance tissue repair primarily by secreting bioactive molecules (immunomodulatory factors, pro-angiogenic factors and anti-apoptotic factors) and microvesicles 6-10; however, how the administered cells adapt to the hostile tissue environment and what paracrine factors they produce locally are largely unknown. Most MSC secretome studies were performed in cell culture, and the identified factors may not represent their true function *in vivo*. Currently, little is known about the MSC proteome in the ischemic heart to suggest their potential reparative activities, and this is largely due to the lack of an effective technique to allow analysis of global scale protein expression in cell-type specific manner.

Analyzing newly synthesized proteins *de novo* is perhaps the most accurate way to understand the functional states of specific cells in a complex tissue. Not until recently has the cell-type specific labeling and isolation of nascent proteomes become available, in which mutant methionyl-tRNA synthetase (MetRS) is targeted to specific cells, enabling incorporation of methionine surrogate azidonorleucine (ANL), a non-canonical amino acid, into newly-synthesized polypeptides 11. The ANL labeled proteins can then be identified by a copper catalyzed azide-alkyne based click reaction for visualization (by bioorthogonal noncanonical amino acid tagging [BONCAT] 12 or fluorescent non-canonical amino-acid tagging [FUNCAT]) 13 or for isolation/enrichment and identification by MS 14. The L274G mutation of the methionyl-tRNA synthetase (MetRS^L274G^) is particularly suitable for mammalian cells and permits ANL incorporation to N-termini and internal sites where the methionine is otherwise located 15.

In this work, we have introduced the MetRS^L274G^ virally into MSCs and administered the transduced cells into ischemic and sham myocardium, then quantitatively analyzed the time-course dynamics of the nascent MSC proteomes by liquid chromatography (LC)/mass spectrometry (MS) and bioinformatics analysis. Our study revealed highly sensitive and specific identification of MSC proteomes with this biorthogonal based labeling and potential novel mediators that underlie MSCs' adaptive responses and reparative functions in the hostile tissue environment.

## Materials and Methods

### MSC culture and characterization

C57BL/6 mouse-derived bone marrow MSCs were purchased from Cyagen Biosciences (Sunnyvale, CA, USA, Catalog Number: MUBMX-01001). The cells were cultured in Dulbecco modified Eagle medium (DMEM) supplemented with 10% fetal bovine serum (FBS) and used for experiments at passage 8 to 10. Their identity was authenticated by characteristic MSC surface marker expression in flow cytometry analysis and by multi-lineage potentiality. The osteogenic and adipose differentiation assays were performed by following manufacturer's protocols (Cyagen Biosciences, USA). Briefly, the cells were initially seeded on 0.1% gelatin-coated 6-well plate (2×10^4^ cells/cm^2^) in complete medium till confluence, then treated with osteogenic differentiation medium for 3 weeks or with adipocyte induction medium and maintenance medium in alternate for 2-3 weeks. The osteogenic differentiation and adipose differentiation were analyzed by Alizarin staining and by Oil Red O staining (for lipid droplet), respectively.

### Flow cytometry

Flow cytometry was performed as we previously described 16. Briefly, 1X10^6^ cells were incubated in 200 μL cold PBS with Fc block (1:100 dilution) and after wash for 3 times, stained with PE-conjugated antibodies for mouse CD34 (1:50) or CD44 (1:50), FITC-conjugated antibodies for mouse CD11b (1:50), CD45 (1:50), or Sca-1 (1:50) (ebioscience, Germany). The data were acquired with BDTM LSR II (BD Biosciences, US) and analyzed with Cell Quest Software (Becton Dickinson, UK).

### Lenti-MetRS^L274G^ vector

pMaRSC plasmid coding for MetRS^L274G^ mutant-mCherry fusion protein was purchased from Addgene (Plasmid #89189). The MetRS^L274G^-mCherry expression cassette (3.6kb fragment) was amplified by high-fidelity PCR using two primers (Forward primer: 5'-ACGTACTAACTAGTACTGCTTACTGGCTTATCGA-3'; Reverse primer: 5'-GTCATAGCGAATTCTAAACGGGCCCTCTAGACT-3'), which contain SpeI or EcoRI restriction site at the end. The PCR product was then digested with SpeI and EcoRI, purified (Gel Extraction Kit, Qiagen), and ligated (T4 DNA ligase, Promega, USA) into the FUGW lentiviral-vector backbone 17 fragment purified from the EcoRI and XbaI restriction digestions with compatible cohesive ends. To pack FUGW-MetRS^L274G^-mCherry lentiviral vector, the plasmid was co-transfected with pMD2.G and psPAX2 in 293T cells as we previously described 18-20. For lentivirus infection, MSCs were seeded at ~80% confluence, and lentivirus was applied with the addition of polybrene (10 μg/mL). 72 h later, the transduction efficiency was evaluated by mCherry expression. Briefly, MSCs were fixed in 4% paraformaldehyde in PBS and permeabilized with 0.1% Triton X-100 for 10 min at room temperature. The primary antibody for mCherry (10 ng/mL, Invitrogen, Waltham, MA, USA) and Alexa fluor 568 conjugated secondary antibody (Life technologies, Waltham, MA, USA) were used for detection of mCherry. The MSCs transduced with FUGW-mCherry lentiviral vector or non-transduced were used as controls.

### Fluorescent non-canonical amino-acid tagging (FUNCAT)

For FUNCAT of MetRS^L274G^-transduced MSCs *in vitro*, the cells were cultured in 1 mM ANL (Jena Bioscience, Jena, Germany) for 24 h, then gently rinsed with PBS, fixed in 3.7% formaldehyde in PBS and permeabilized with 0.5% Triton X-100 in PBS. Click-iT reaction was performed with Click-iT reaction cocktails containing Alexa Fluor 488 alkyne (Click-iT Alexa Fluor 488 Protein Synthesis HCS Assay kit, Life Technologies, Waltham, MA, USA) according to the manufacturer's protocol. Cells were stained with Hoechst 33342 prior to imaging. For FUNCAT of MetRS^L274G^-transduced MSCs administered in the mouse heart, ANL was i.p. administrated at 15 mg/kg every 6 h for 4 times, then the animal was euthanized and the heart was isolated and immediately placed in pre-cooled 4% paraformaldehyde on ice. After overnight at 4 °C, the tissue was dehydrated in 30% sucrose for 24 h and sectioned. The tissue sections were then evaluated by expression and Click-iT reaction as described above.

### Bio-orthogonal non-canonical amino acid tagging (BONCAT)

After incubation in 1 mM ANL for 24 h, MetRS^L274G^-transduced MSCs were lysed by RIPA buffer supplemented with protease inhibitors (Sigma, USA) and then, total proteins were collected. Click-iT reaction was performed following the instruction of Click-iT Protein Reaction Buffer Kit (Invitrogen, Waltham, MA, USA). Briefly, up to 200 μg of azide-labeled protein were reacted with 100 μL of Click-iT reaction buffer containing alkyne-Cy7 in a rotator for 20 min, under the catalyzation of copper sulfate. Methanol and chloroform were then used to remove residual reaction components and precipitate proteins. The precipitated protein samples were solubilized in laemmli buffer and loaded for gel electrophoresis. Gels were then immediately imaged under stain free gel mode or Cy7 mode using a ChemiDoc MP Imaging System (Bio-Rad, California, USA).

### Mouse care, acute MI surgery and MSC administration

All animal experiments in this report were approved by the Institutional Animal Care and Use Committee (IACUC) of the University of Alabama at Birmingham and performed in compliance with the “Guide for the Care and Use of Laboratory Animals” (NIH publication). 8-10 weeks old C57BL/6J mice (Jackson Laboratory, Bar Harbor, ME, USA) were used for all the experiments unless specified. For surgical induction of AMI, mice (n=21) were anesthetized with inhaled isofluorane (1.5-2%), intubated and ventilated; then, a left thoracotomy was performed, and AMI was induced by permanent ligation of the left anterior descending coronary artery (LAD) as we previously described 21. The Sham-operated animals (n=18) went through all the procedures except LAD ligation. Each animal received 5×10^5^ MetRS^L274G^ MSCs or control virus-infected MSCs (diluted in PBS) injected intramyocardially at three different sites in the ischemic area, using a 30-gauge needle. The chest was subsequently closed and the mice were allowed to recover in heating pad before return to animal holding room. The post-surgery care and pain management were performed according to our IACUC-approved protocol. For pain management, the mice were injected subcutaneously with Buprenex (0.05-0.2 mg/kg) every 12 h for 2 d and with Metacam (1 mg/kg) daily for 3 d after the surgery. Three mice in the MI group died one day after the surgery (mortality rate: 14%). The mice were euthanized at different time points by CO_2_ inhalation followed by vital organ (i.e., heart) removal.

### Isolation of ANL-labeled proteins from MetRS^L274G^ MSCs-transplanted hearts

For labeling of MSC proteome from cardiac tissue, ANL was i.p. administrated (15 mg/kg) at day 0, 2, and 6 after MI surgery and cell injection, once every 6 h for 4 times; at day 1, 3, or 7, the mice were euthanized and left anterior wall of the hearts were isolated and subjected to Click-iT reaction (Click-iT Protein Enrichment Kit, Invitrogen, Waltham, MA, USA) for capture of ANL-labeled proteins on an alkyne resin according to the manufacturer's instructions (n=5-6 per time point per group). Specifically, the collected heart samples were incubated in the lysis buffer containing 8 M urea, 200 mM Tris pH 8, 4% CHAPS, 1M NaCl and 1% Protease inhibitor (Sigma, Burlington, MA, USA), followed by sonication on ice. After a centrifugation at 10,000 g at 4 °C for 5 min, the supernatants were collected and incubated with reaction solution (containing copper sulfate) and resin slurry at room temperature for 18 h in an end-over-end rotator. The protein-binding resin was then pelleted and reduced in 10 mM DTT/SDS buffer by heating at 70 °C for 15 min and cooled at room temperature for 15 min. After reduction, the resin was further pelleted, resuspended in 7.4 mg/mL iodoacetamine (Sigma, Burlington, MA, USA) /SDS buffer and incubated in the dark for 30 min for eliminating background reaction. Subsequently the resin was washed with SDS buffer, 8 M urea and 20% acetonitrile through a spin column to achieve stringent removal of non-specifically bound proteins. After digesting the resin with 100 mM Tris, 2 mM CaCl_2_ and 10% acetonitrile, the resin-bound proteins were further digested in 0.1 µg/µL MS-grade trypsin (Thermo Fisher Scientific, Waltham, MA, USA) at 37 °C overnight. The digested proteins were then desalted on a C-18 cartridge (Waters, Milford, MA, USA, catalog number: WAT036820), dried in a vacuum concentrator (SpeedVac) and stored at -20 °C for liquid-chromatography-coupled tandem mass spectrometry (LC/MS).

### Sample preparation and data acquisition by LC-MS

Dried peptides were reconstituted in 16 µL of 0.1% formic acid (FA). 8 µL of each sample was injected onto a 1260 Infinity nHPLC stack (Agilent Technologies, Santa Clara, CA, USA), and separated using a 75 micron I.D. × 15 cm pulled tip C-18 column (Jupiter C-18 300 Å, 5 micron, Phenomenex). This system runs in-line with a Thermo Orbitrap Velos Pro hybrid mass spectrometer, equipped with a nano-electrospray source (Thermo Fisher Scientific, Waltham, MA, USA), and all data were collected in CID mode. The nHPLC was configured with binary mobile phases that included solvent A (0.1%FA in ddH2O), and solvent B (0.1% FA in 15% ddH_2_O / 85% ACN), programmed as follows; 10 min at 2% solvent B; 90 min at 5-40% solvent B; 5 min at 70% solvent B; 10 min at 0% solvent B. Following each parent ion scan (300-1200m/z @ 60k resolution), fragmentation data (MS2) was collected on the top most intense 15 ions. For data dependent scans, charge state screening and dynamic exclusion were enabled with a repeat count of 2, repeat duration of 30 s, and exclusion duration of 90 s.

### MS data conversion and searches

The XCalibur RAW files were collected in profile mode, centroided and converted to MzXML using ReAdW v. 3.5.1. The mgf files were then created using MzXML2Search (included in TPP v. 3.5) for all scans. The data was searched using SEQUEST, which was set for two maximum missed cleavages, a precursor mass window of 20 ppm, trypsin digestion, variable modification C @ 57.0293, and M @ 15.9949. Searches were performed with a specific subset of the UniRef100 database.

### Peptide filtering, grouping, and quantification

The lists of peptide IDs generated based on SEQUEST (Thermo Fisher Scientific, Waltham, MA, USA) search results were filtered using Scaffold (Protein Sciences, Portland, Oregon, USA). Scaffold filters and groups all peptides to generate and retain only high confidence IDs while also generating normalized spectral counts across all samples for the purpose of relative quantification. Only peptides with charge state of ≥2+, a minimum peptide length of six amino acids, and nonzero quantities for all six mass tags were accepted for analysis. Scaffold incorporates the two most common methods for statistical validation of large proteome data sets, false discovery rate (FDR), and protein probability. The FDR was set at <1% cutoff, with an individual peptide probabilities ≥0.8, protein probabilities of ≥0.99, and at least two peptides assigned per protein. Relative quantification was performed via spectral counting and spectral count abundances were normalized between samples.

### Bioinformatics and statistics analysis

For the protein abundance data, Pearson correlation was used to evaluate the protein expression correlation between samples. Hierarchical clustering was performed to cluster samples and proteins profiles based on complete linkage method. Differential protein expression between the MI and Sham groups was assessed by Mann Whitney U-test for each time point, i.e., day 1, day 3 and day 7. The lists of differentially expressed proteins (DEPs) were obtained by the criteria of nominal *p* value <5% and fold change cutoff >1.5 and visualized by volcano plots and heatmaps. For those proteins that were detected in ≥60% of one group but none in the other group, we followed the method by Kojima et al 22 with a more stringent cutoff of over 60% presence in only one group and defined them as “all-or-nothing” protein hits, which were also included in the lists of DEPs. To identify the significantly enriched functional pathways, the lists of DEPs were analyzed with multiple databases (KEGG, Reactome, Panther and Wikipathay) using the WebGestalt platform (http://www.webgestalt.org/) 23. Pathway enrichment plots were subsequently made with the R platform. The Gene Ontology (GO) analyses were performed with R Bioconductor package, topGO, to identify the GO biological processes and cellular components that are significantly associated with the individual lists of DEPs. The protein-protein interaction network analysis was made with the software STRING (https://string-db.org/) 24, followed by the visualization with the package Cytoscape 25.

## Results

### MetRS^L274G^ expression allows ANL labeling of newly synthesized proteins in the MSCs

**Figure 1:**
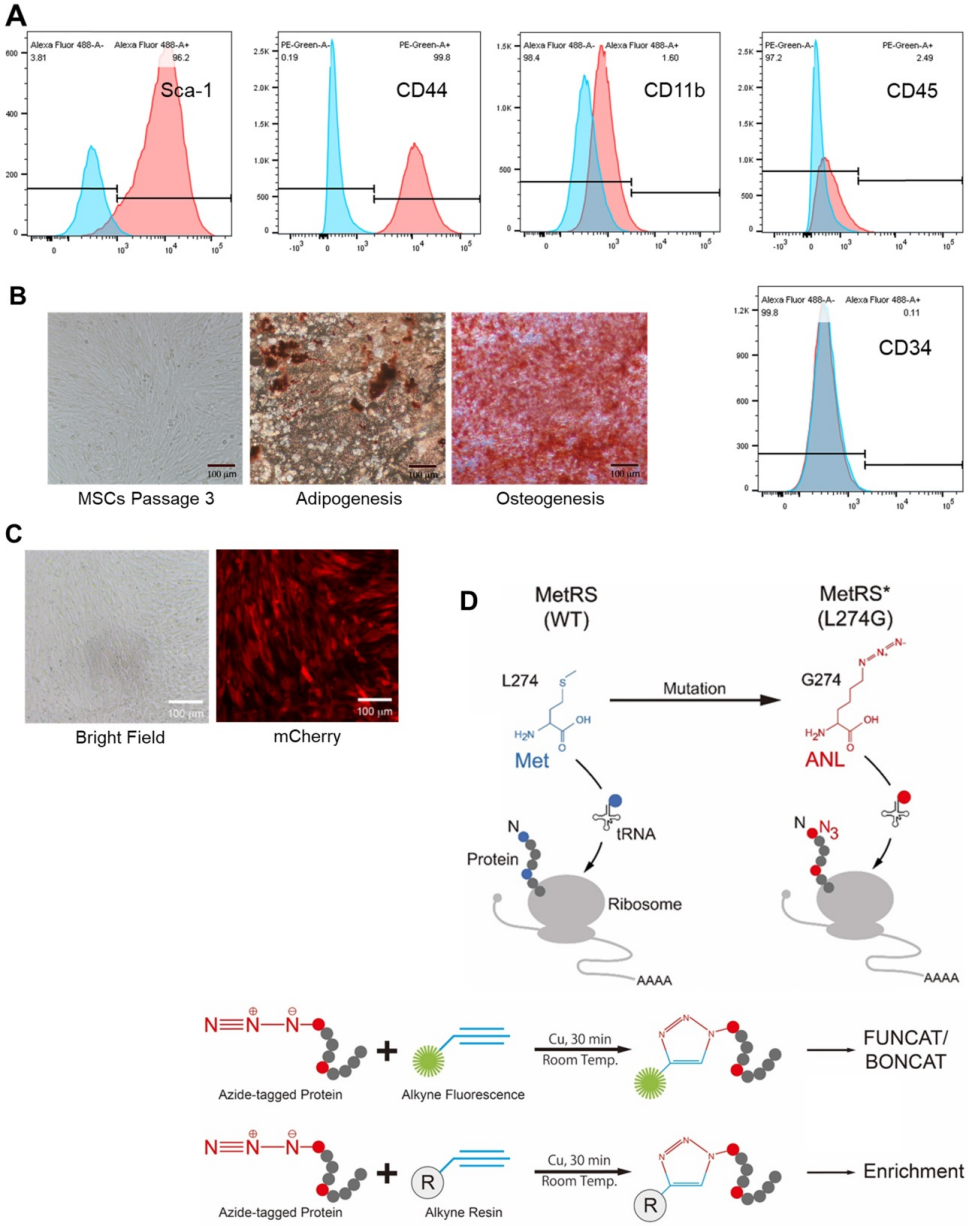
** Characterization, genetic modification and proteome-labelling strategy of MSCs.** (**A**) FACS analysis of MSCs for cell surface markers. (**B**) MSCs under a phase-contrast microscope (*Left* panel) and after differentiation in culture into adipocytes (*Middle* panel, oil red staining) and osteocytes (*Right* panel, alizarin red staining). Shown are representatives of three independent experiments. (**C**) MSCs were infected with lentiviral vector MetRS^L274G^-mCherry, and the transduced cells were FACS-selected by mCherry expression. (**D**) Expression of MetRS^L274G^ allows loading of the methionine surrogate azidonorleucine (ANL) onto methionine tRNA, and subsequently ANL incorporation in newly-synthesized proteins (*Upper* panel). The ANL-tagged proteins are clicked to alkyne-containing dye or resin in the presence of copper, and detected by FUNCAT/BONCAT or enriched for LC-MS (*Lower* panel).

**Figure 2:**
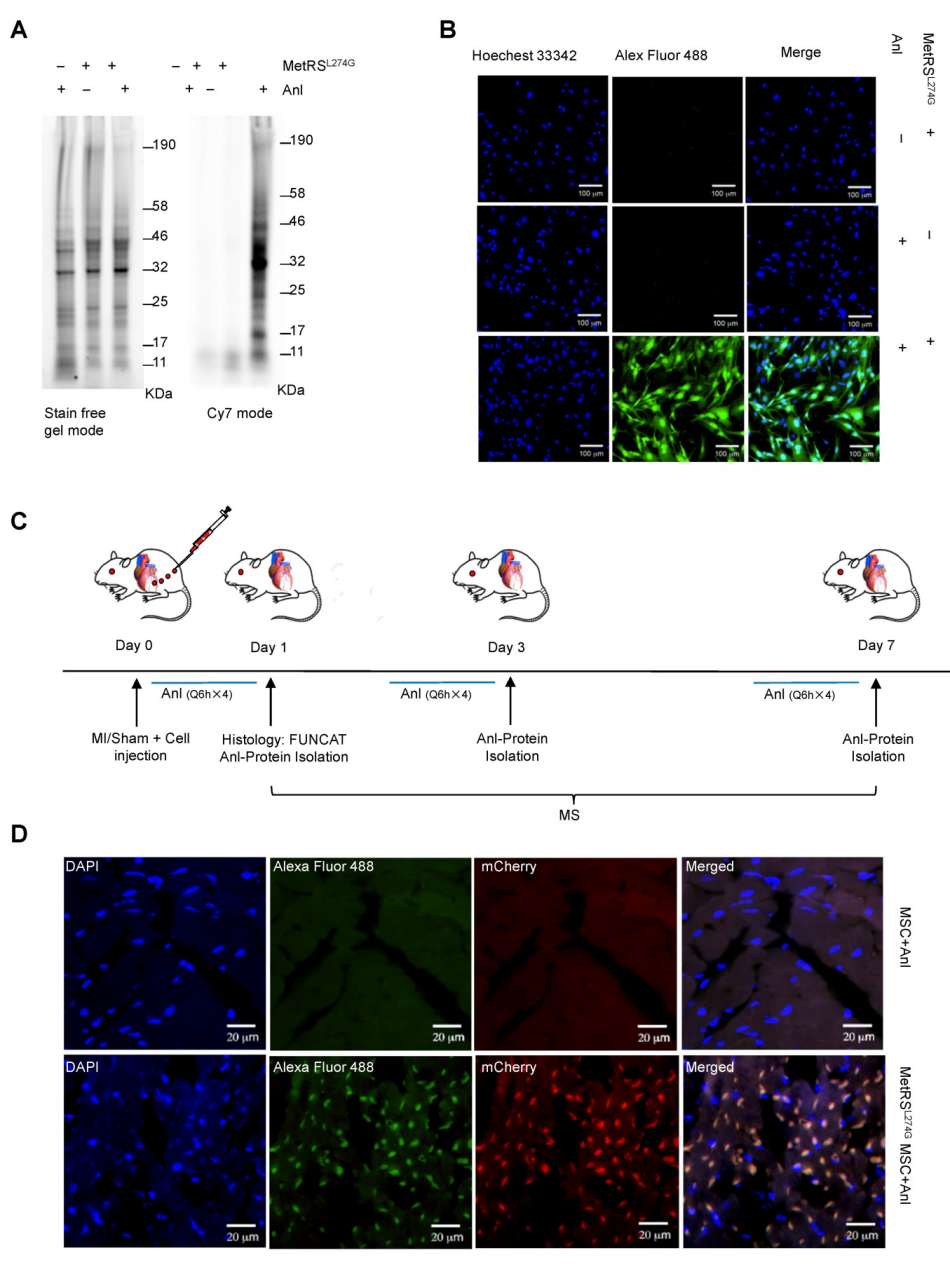
** MetRS^L274G^ expression allows ANL labeling of newly synthesized proteome of MSCs.** (**A-B**) The MetRS^L274G^-mCherry (+) or control virus (-) transduced MSCs were incubated with ANL (+) or vehicle (-) for 24 h. (**A**) BONCAT was performed in cell lysates to stain the ANL-incorporated nascent protein using Click-iT protein reaction kit (alkyne-Cy7) and imaged in SDS-PAGE gel in Cy7 mode, with stain-free gel mode as loading control. (**B**) FUNCAT was performed to visualize MSCs containing ANL-tagged proteins. (**C**) Workflow for analysis of MSC proteome *in situ* in the ischemic myocardium. The mice were subjected to MI or Sham surgery and immediate intramyocardial injections of MetRS^L274G^-mCherry transduced MSCs in the infarct border area, then randomized to receive i.p. injections of ANL (Q6h X 4) at day 0, 2, or 6, and euthanized 24 h later (at day 1, 3, or 7) for histological and biochemical analysis of tissues. n=5-6 mice for each time point in each group. (**D**) Representative FUNCAT in the MSCs administered in the myocardium for 1 d. Red, mCherry; Green, alkyne-Alexa Fluor 488.

We obtained murine MSCs from commercial source and verified their identity by the expression of characteristic surface markers with flow cytometry; MSCs are homogenously positive for Sca-1 and CD44 but negative for CD45 and CD34 (**Figure 1A**). These cells displayed a uniform spindle-shaped morphology under a phase-contrast microscope and were robustly induced to differentiate into adipocytes and osteocytes (**Figure 1B**). To enable non-canonical amino-acid tagging in MSCs, we generated a MetRS^L274G^-mCherry lentiviral vector (**Figure S1**); one infection results in the majority of MSCs to express mCherry, and the stably transduced cells were further selected by FACS (**Figure 1C**). Then, the cells were treated with 1 mM ANL for 24 h to allow incorporation into the newly-synthesized proteins, then lysed, and the proteins extracts were subjected to click-iT reaction (BONCAT) for detection of ANL labeling (**Figure 1D**). Abundant ANL-labeled proteins were observed in the lenti-MetRS^L274G^-transduced and ANL-treated MSCs, spanning all molecular weights (**Figure 2A**). In contrast, no ANL-labeled proteins were detected in the controls, virus-infected/vehicle-treated cells or control virus-infected/ANL-treated cells. We also stained ANL-labeled proteins in the lentivirus transduced cells by alkyne conjugated with Alexa Fluor 488 (FUNCAT). The ANL-labeled proteins were intensively stained in the ANL-treated MetRS^L274G^ MSCs, with no signal detected in the control groups of cells (**Figure 2B**). Thus, transduction of MetRS^L274G^ allows sensitive and specific identification of ANL-labeled proteins by click reaction.

### Detection of ANL labeling of the MetRS^L274G^-transdued MSCs in the heart

To investigate the dynamic expression of MSC proteomes in the ischemic and normal hearts, we administered MetRS^L274G^-transduced MSCs into the infarct border area in the MI mice or into the comparative area in the Sham mice immediately after the surgery, then i.p. injected ANL (15 mg/kg) starting at 3 different time points (day 0, 2, or 6 post-surgery/cell administration), every 6 h for 4 times, then harvested the heart samples at day 1, 3, or 7, respectively, to prepare left ventricles (LV) tissue slices and cell lysates (**Figure 2C**). The tissue slices were subjected to click-iT reactions with alkyne-Alexa Fluor 488 (FUNCAT), and the MetRS^L274G^ expression in all the transplanted MSCs resulted in a strong FUNCAT signal, indicating nascent ANL-tagged proteins in these cells (**Figure 2D**). In contrast, no protein was labeled in the administered control virus-transduced MSCs despite ANL was also provided, which indicates that the protein labeling method is highly sensitive and selective *in vivo* (**Figure 2D**).

### Isolation and analysis of the MSC proteome in the MI vs. Sham hearts at different time points

**Figure 3:**
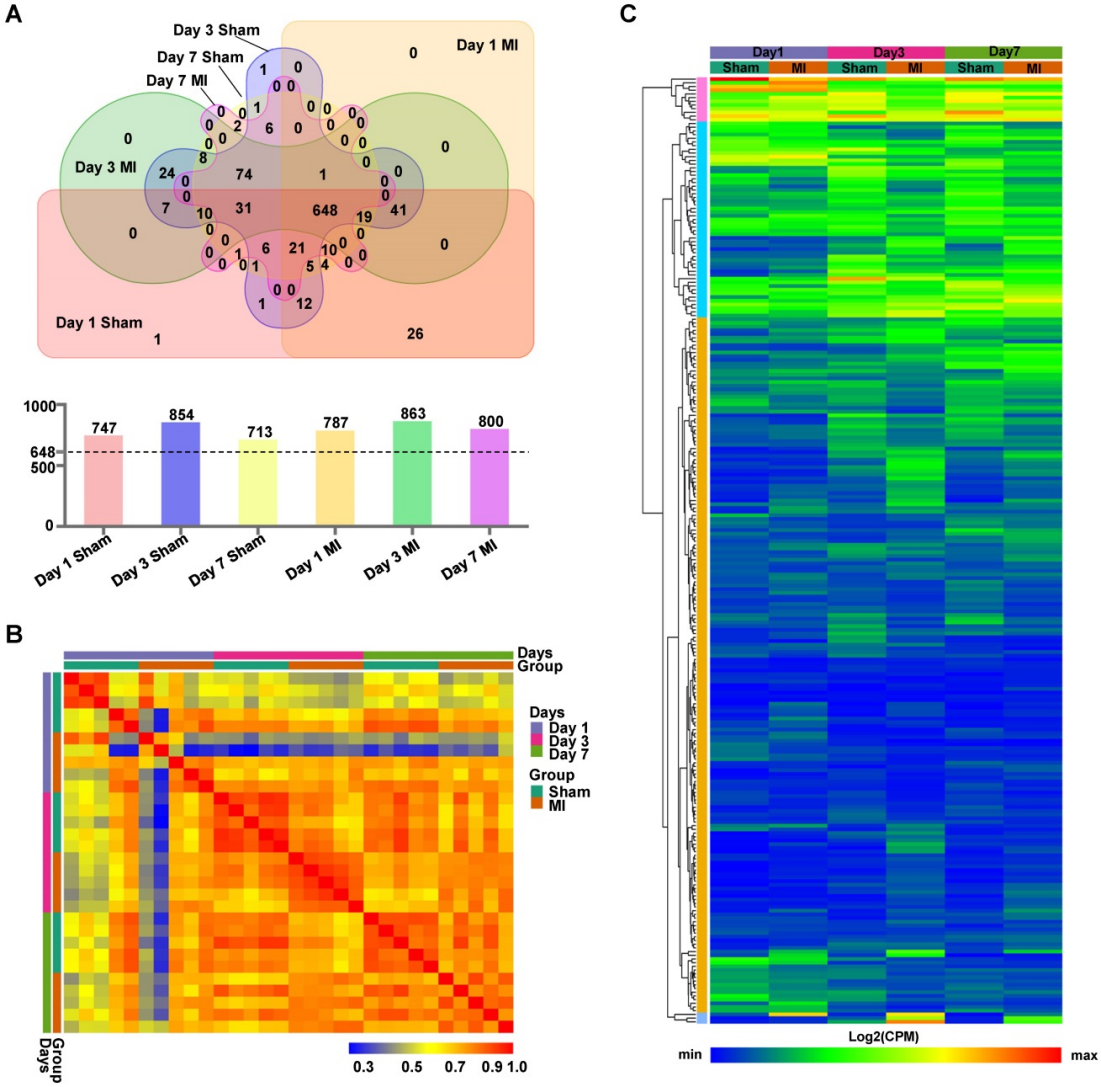
** Identification of MSC-specific proteome in the heart.** (**A**) Venn diagram analysis of proteomes in the six experimental groups, Day 1 MI, Day 3 MI, Day 7 MI, Day 1 Sham, Day 3 Sham, and Day 7 Sham, showing numbers of proteins unique to each group or shared by different groups. There were 713 - 863 proteins identified in the six groups (*upper* panel), and 648 proteins common to all groups account for 77~87% of the total proteins for individual groups (*lower* panel). (**B**) Pearson correlations between samples within each group at day 1, day 3 and day 7. (**C**) Hierarchical clustering of proteins expressed in all groups. Each column corresponds to the mean expression levels of proteins in 5-6 independent biological samples, and each row corresponds to a protein.

The LV cell lysates, comprised of five biologically independent samples for each group at each time point, were subjected to isolation of ANL-labeled proteins by clicking into a cleavable alkyne-tag immobilized on resin and subsequently to LC/MS analysis (**Figure S2**). We routinely obtained ~4 μg of ANL-labeled proteins from each mouse heart transplanted with 5×10^5^ MSCs, which accounts for ~0.8% of total proteins from the donor cells. Notably, a relatively large number of ANL-labeled proteins were identified in the MetRS^L274G^ MSC-injected Sham and MI hearts at days 1, 3, and 7 post-surgery/cell administration (747, 854, and 713 for Sham and 787, 863, 800 for MI, respectively). Interestingly, 648 proteins were shared among all groups, accounting for 82±5% of all identified proteins in individual group (**Figure 3A**). Pearson correlation analysis revealed that the expression levels of proteins were consistent within each group, indicating high reproducibility of our method (**Figure 3B**). Then we performed unsupervised hierarchical clustering of protein samples expressed in all groups. As expected, there is a substantial common pattern of expression among different groups, but our results also reveal the proteomes between Sham and MI group are quite distinguishable due to many DEPs (**Figure 3C**).

To gain a better understanding of the functional proteome, we performed GO analyses of all identified proteins in each group at the three time-points for cellular components, biological process and molecular function. In both Sham and MI hearts of different time points, the identified proteins that show the highest fold enrichment are under the terms of mitochondrion, extracellular exosomes, oxidation-reduction process and poly(A) RNA binding (**Figures S3 and S4**); this is not surprising given that these terms represent fundamental cellular process, and that the majority of the identified proteins are shared by all groups; rather, these results suggest high fidelity of this protein identification technique.

### The differentially expressed MSC proteins in MI vs. Sham hearts are more pronounced in extracellular matrix organization, response to stress and regulation of apoptotic process

**Figure 4:**
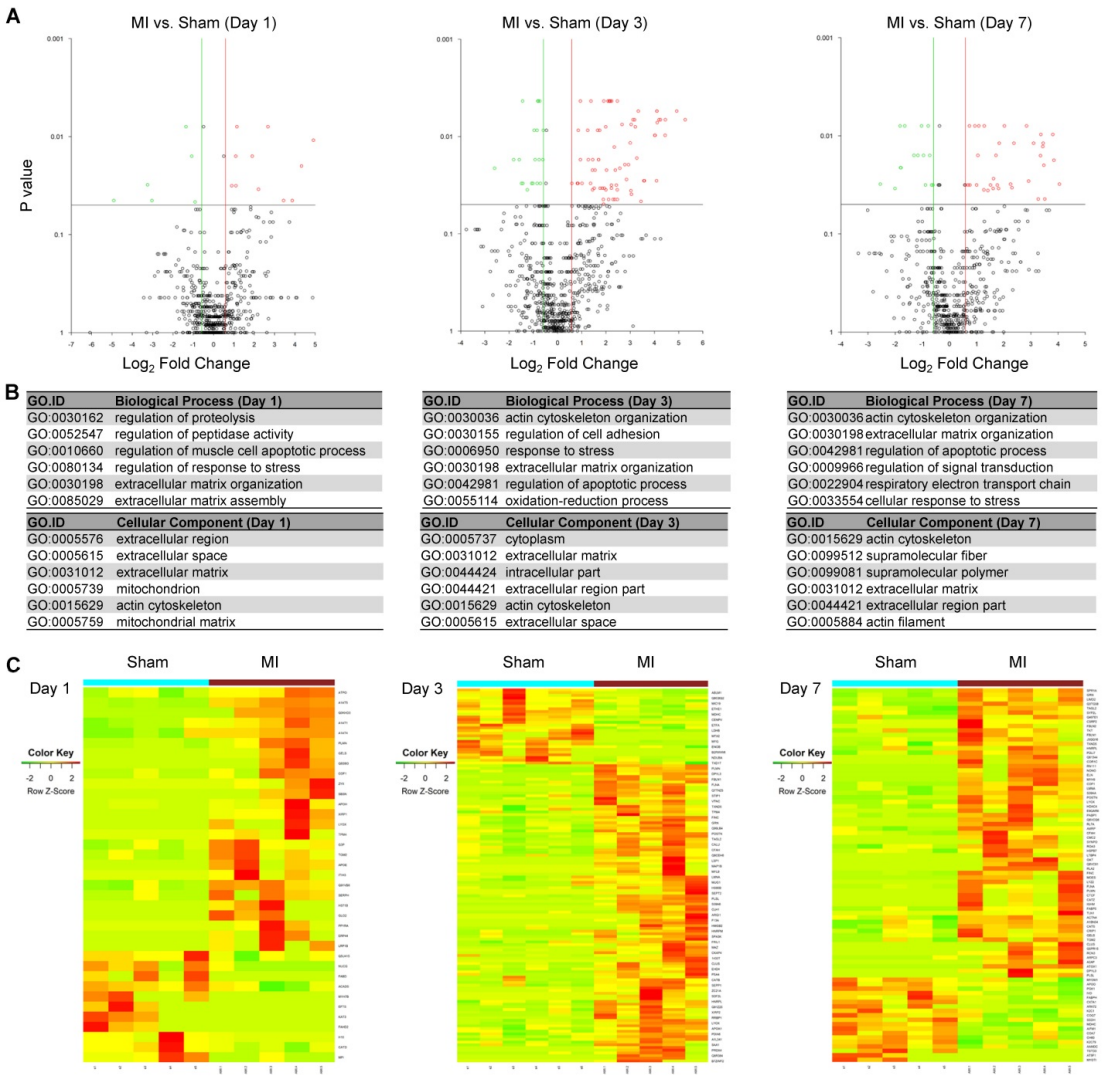
** Identification of DEPs from the MSCs in MI vs. in Sham heart.** (**A**) Volcano plots showing DEPs from the MSCs in MI vs. in Sham heart at day 1 (*left* panel), day 3 (*middle* panel) and day 7 (*right* panel) after surgery and cell administration. Each point represents the difference of fold-change (X axis, Log2[fold change]) plotted against the level of statistical significance (Y axis, p value). Red dots indicate upregulated proteins, and green dots indicate downregulated proteins. *p* value was calculated by Mann Whitney U-test, ratio >1.5 or < 0.67. n=5 animals per group. (**B**) GO analyses of DEPs under the terms of cellular components, biological process. *p* value was calculated by Mann Whitney U-test, ratio >1.5 or < 0.67. (**C**) Heatmaps showing DEPs from the MSCs in MI vs. in Sham heart at day 1 (*left* panel), day 3 (*middle* panel) and day 7 (*right* panel). n=5-6 animals per group.

To identify differentially expressed MSC proteins in ischemic vs. normal hearts, we compared their profiles by volcano plot. Interestingly, 26, 110 and 65 proteins were significantly up-regulated while 11, 28 and 19 proteins were significantly down-regulated in MI vs. Sham hearts at day 1, 3 and 7, respectively (Fold change ≥2; **Figure 4A** and **Figure S5-S7**). The significantly DEPs are enriched under GO terms extracellular matrix organization (cofilin-1, filamin-A, fibronectin), response to stress (HSP90, protein S100A9, HMGB2, complement factor H) and regulation of apoptotic process (cathepsin, prelamin-A/C, mitochondrial apoptosis-inducing factor 1) (**Figure 4B**), which likely reflect how MSCs respond to ischemic/hypoxic myocardial environment and exert their effects on cardiac repair. To better visualize and interpret the data, we presented the levels of individual DEPs in heatmap (**Figure 4C**) and bar graphs (**Table S1**). For example, Filamin-A increased by 12 folds in the cells in MI heats from day 1 to day 3, while by just 4 folds in those in Sham hearts. Interestingly, cofilin-1 and fibronectin were detected at a higher level in the cells of MI group than in Sham group, which correlates with the deposition of fibrotic tissue. These data suggest that the MSCs' effects on ischemic hearts may be largely attributable to their production of extracellular matrix. In addition, a number of DAMPs, including HSPs, protein S100A9, HMGB2, complement factor H, was up-regulated, indicating the involvement of MSCs in the regulation of inflammation. Collectively, these results suggest that the identified proteomes reflect the pathological status of MSCs in the ischemic hearts and further validate the MetRS labeling and enrichment technique that sensitively captured adaptive changes in MSC proteome *in situ*.

### MSCs respond to the ischemic/hypoxic environment by altering proteins in the complements and coagulation cascades and regulators of actin cytoskeleton and apoptosis

**Figure 5:**
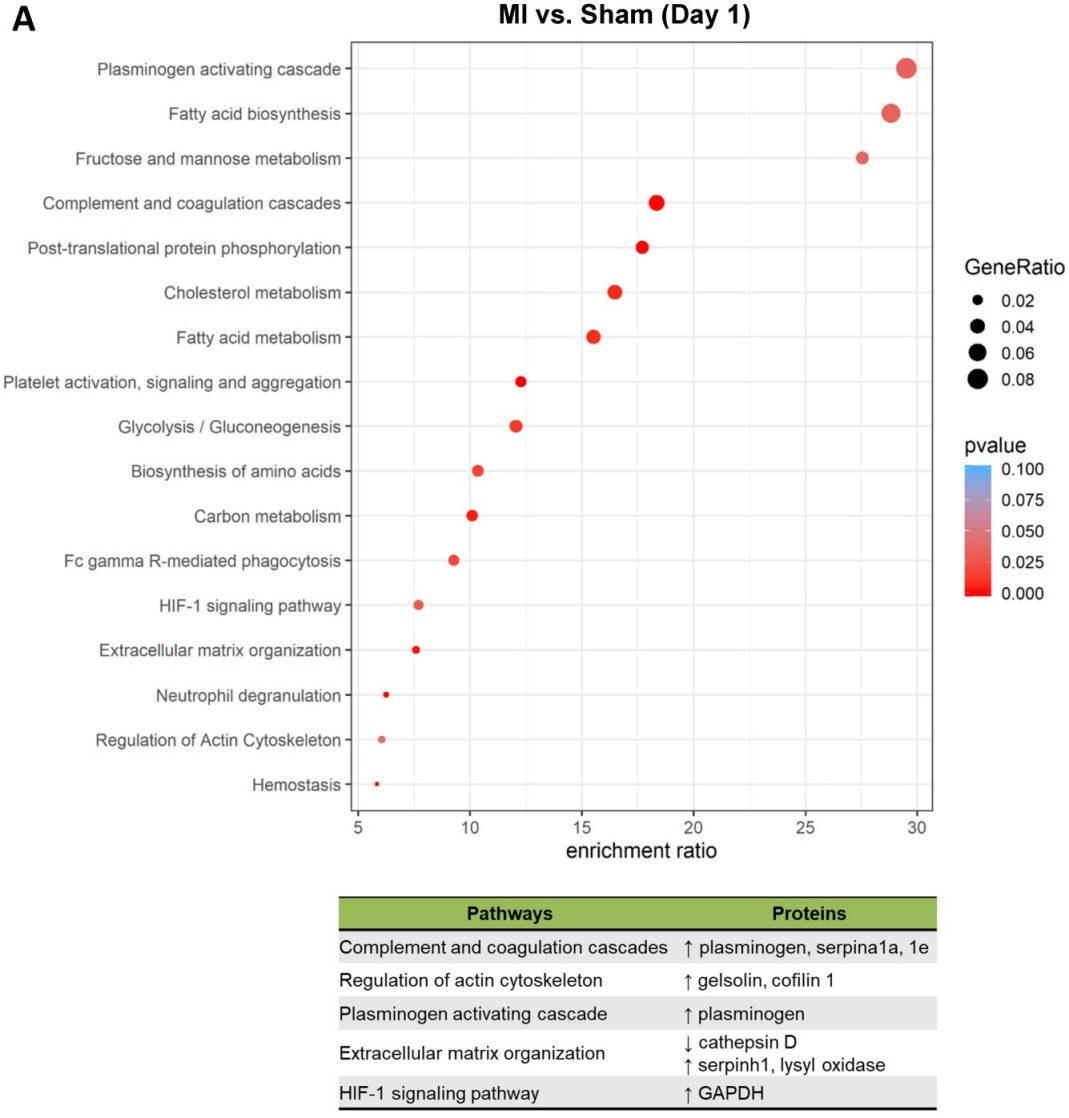

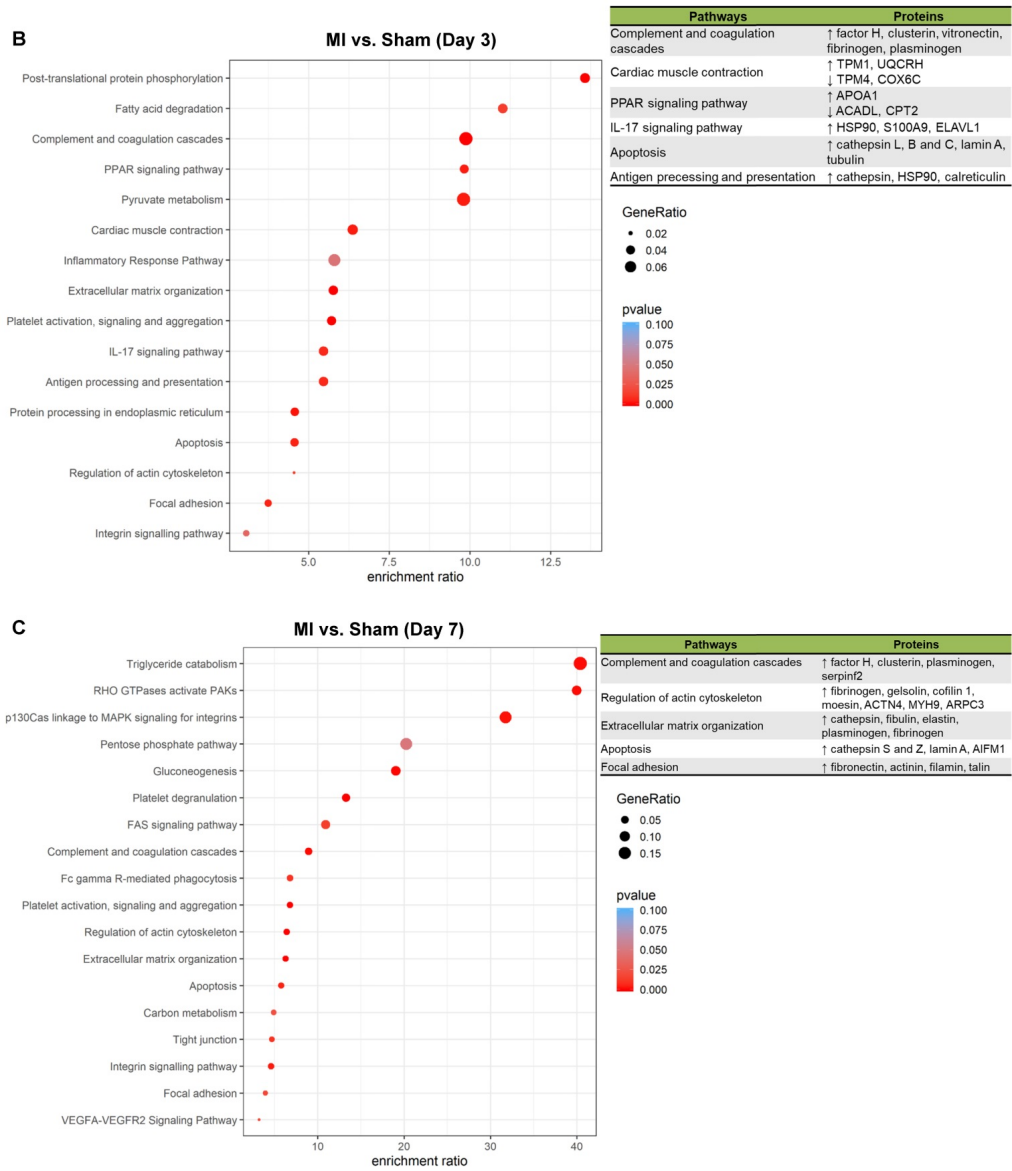
** Pathway analysis of DEPs from the MSCs in MI vs. in Sham heart at serial time points.** (**A-C**, *upper* panels): KEGG enrichment analyses of DEPs from the MSCs in MI vs. in Sham heart at day 1 (**A**), day 3 (**B**), and day 7 (**C**). The most significantly enriched KEGG pathways are illustrated with bubbles, with X axis indicating the enrichment factor (i.e., the ratio of the count of the DEPs observed in a certain pathway vs the count of proteins expected by random chance in the same pathway), Y axis indicating the enriched pathways, bubble size indicating the number of proteins enriched in the corresponding pathway, and bubble color indicating Q value (adjusted p value) of the corresponding pathway. (**A-C**, *lower* panels) Individual proteins enriched in the analyses with potential biological effects in MSC reparative function are listed under corresponding pathways.

To better understand the functional states of MSCs in the ischemic myocardium, we performed Kyoto Encyclopedia of Genes and Genomes (KEGG) pathway analysis and KEGG enrichment analysis of differentially expressed proteins. At day 1, pathways and proteins related to complement and coagulation cascades, regulation of actin cytoskeleton, and extracellular matrix organization were highly enriched in the MSCs in the MI hearts as compared to those in Sham hearts (**Figure 5A**; *upper* panel, analyses; *lower* panel, individual pathways and proteins). Remarkably, these pathways remain enriched throughout day 3 and day 7 (**Figure 5B-C**). Interestingly, we also found additional enriched pathways, including apoptosis, antigen processing and presentation and IL-17 signaling pathway for day 3 (**Figure 5B**) and apoptosis and focal adhesion for day 7 (**Figure 5C**). Notably, proteins enriched for complement and coagulation cascades (factor H, clusterin and plasminogen) are reportedly associated with inflammation 26, 27 and cardiac regeneration 28, 29, suggesting that MSC proteome and secretome may be a valuable resource for therapeutics of heart ischemia. The abundant proteins enriched for regulation of actin cytoskeleton and apoptosis reflect the *in vivo* characteristics of MSCs under ischemia to a significant extent 30.

**Figure 6:**
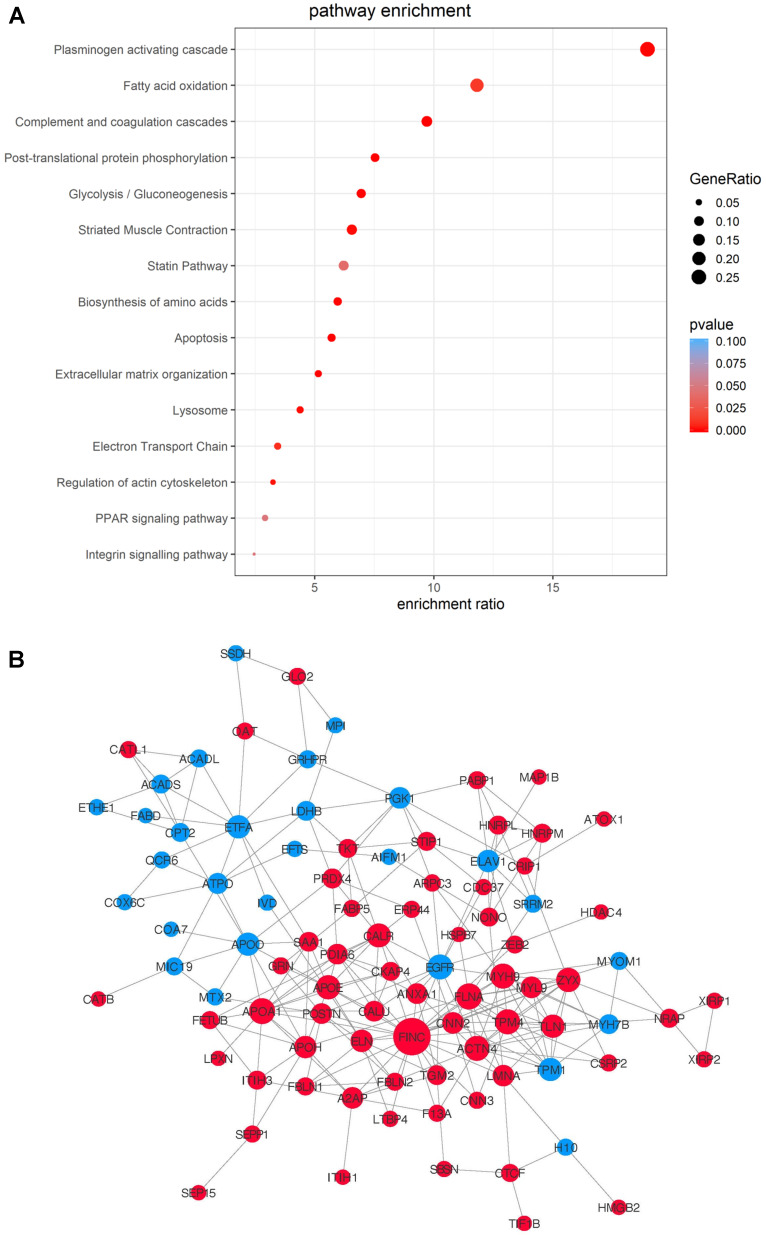
** Integrated analysis of DEPs from the MSCs in MI vs. in Sham heart with combined time points.** Data of the three time-points within each treatment group, MI vs. Sham, were combined for differential MSC proteome analyses. (**A**) Pathway enrichment analyses. (**B**) The protein-protein interaction network analyses with nodes indicating individual proteins, edges indicating interactions between connecting proteins. The degree of a node indicates the number of connections to other nodes (i.e., the potential importance as protein-protein interaction hubs), edge thickness indicates the degree of interactions, and the color of nodes denotes up (red) or down (blue) regulation.

To gain an overall understanding of MSC-mediated ischemic response in cell therapy and to take into account of the proteins' functional interactions, we integrated the data from the three time-points and compared the potential pathways and networks of the differentially-expressed proteins in MI vs. Sham hearts. The transplanted cells, regardless of the time points, showed massive proteome remodeling characterized by pathways related to complement and coagulation cascades, plasminogen activating cascade, apoptosis, extracellular matrix organization, lysosome, regulation of actin cytoskeleton, PPAR signaling pathway following MI (**Figure 6A**). The pathway of plasminogen activating cascade possesses the highest number of proteins with the most significant difference (**Figure 6A**). A protein-protein interaction network was extracted by matching all the DEPs against the STRING-db server (http://string.embl.de/), offering a more detailed topological assessment of proteins interactions (**Figure 6B**). In addition to the marked up-regulation of the complement and coagulation cascades pathway, MSCs in MI express an abundance of proteins related to regulation of actin cytoskeleton (e.g., COF1, FINC, ACTN4, GELS, and ARP2/3) and apoptosis (CTAB, LMNA and AIFM1) (**Figure 6B**), which reflects the functional states of MSCs in adaptation to the MI environment by maintaining cell structure and survival. Notably, proteins involved in inflammation (HS90B, HSPB7, S100A9, HMGB2) are globally up-regulated in the MI hearts (**Figure 6B**). Fibronectin is the highest among the all deferentially expressed genes, thus may serve as an important hub coordinating crucial biological activities of MSCs in the acutely infarcted heart.

## Discussion

To address the ongoing challenge for a better mechanistic understanding of MSC therapy for ischemic heart disease, we adopted a genetic engineering and click chemistry approach to identify proteome dynamics of MSCs in the ischemic heart. Specifically, we targeted MetRS^L274G^ mutation into MSCs to tag nascent proteins with biorthogonal non-canonical amino-acid tagging, profiled their proteomes *in situ* in the MI and Sham hearts. Our results suggest that the technique allows sensitive and specific identification of MSC proteomes *in vivo* and revealed previously unknown molecular players and adaptive changes expressed in the transplanted MSCs. Diverging from MSCs' traditional reparative mechanisms, our findings suggest a role of MSCs in early inflammation post-MI and the source of complement and coagulation cascades in cardiac repair.

The approach to selectively labeling nascent proteins in specified cell types to enable unambiguous determination of donor cell-derived proteins in complex multicellular system *in vivo* is of high biological significance. The success of MetRS^L274G^ in identifying proteomes *in vivo* has recently been independently reported in neuronal cells 15. In our study, MSCs carrying the MetRS^L274G^ mutation could efficiently take up ANL and utilize it in its protein synthesis both at the cell level and at the tissue level. *In situ* proteomes of cells have been pulled out with high efficiency and selectivity from each dissected heart sample with most proteins reflecting the *in vivo* characteristics of MSCs in the tissue. Thus, this technique provides a unique opportunity to measure proteomes in modified cell types within the intact pathophysiological system in an accurate and sensitive way and minimally affect protein function. Importantly, this method allows for accurate identification of MSC proteomes *in situ* without mechanical dissociation of the transplanted cells.

Cardiac repair after MI initiates with an early phase of inflammation characterized by immune cell infiltration to eliminate damaged cells and extracellular matrix and recruit reparative cells, followed by a reparative phase characterized by resolution of inflammation, scar formation, and neovascularization 4. Notably, DAMPs are involved in the inflammatory phase by binding to pattern recognition receptors (PRRs) on surviving parenchymal cells and infiltrating leukocytes to activate the complement pathway and a cascade of inflammatory mediators. 31 In this study, we identified a broad upregulation of well-known DAMPs in the MSCs transplanted in the MI heart, including HMGB2, S100 proteins, and several cytokines and chemokines, which suggests MSCs' potential involvement in the early stage of inflammation. It is known that, amongst other modulatory effects of MSCs, the pro-inflammatory effect of MSCs is beneficial during early phases of inflammation whereas their anti-inflammatory effects are useful during later phases when excessive immune activation would cause tissue damage 32.

Diverging from the traditional reparative mechanisms of MSCs, our study highlights the contribution of complement and coagulation cascades from MSCs to cardiac repair after MI. The enhanced expression of complements following MI have been observed for a long time 33, and the temporal regulated activation and suppression of innate immunity was shown to be critical for minimizing myocardial necrosis and optimizing cardiac repair 34. The beneficial effects of complement action under hypoxia include waste disposal, recruitment of stem cells, regeneration, angiogenesis, and better utilization of energy sources 35. More recent research suggests some complements are evolutionarily conserved and actually promote tissue regeneration 29. Our study for the first time revealed that complements are at least partially attributable by transplanted MSCs, which may underline the importance of MSCs in tissue repair. Specifically our study revealed that factor H is contributed by MSCs. Others reported that factor H is necessary to preserve myocardium and myocardial function in chronic MI and promote tissue healing 28, 36, 37. Thus, to delicately modulate, rather than to abruptly blunt, complement activation may be an approach for cardiac repair. How the MSC-derived complements coordinate tissue inflammation vs. regeneration for tissue repair warrant further investigation.

Actin cytoskeleton regulation is critical for cell migration, homing and retention, survival and proliferation, which are key cellular functions for their reparative activity. We observed that proteins involved in regulation of actin cytoskeleton, including cofilin and fibronectin, were upregulated in MSCs in the ischemic heart. Cofilin is a potent actin-binding protein that severs and depolymerizes actin filaments in order to generate the dynamics of the actin cytoskeleton. For example, cofilin phosphorylation promotes the accumulation of stress-like fibers and severely impaires cardiomyocyte contractility 38. As a cytoskeleton-associated protein, Cofilin-1 can also serve as a novel pathway for VEGF-stimulated endothelial cell migration 39 and essential for hypoxia-induced neovascularization 40. Importantly, fibronectins from different sources contribute differentially to specific aspects of angiogenesis 41 and is required for self-renewal and migration of stem cells 42, 43; thus, highly-expressed fibronectin in MSCs under ischemia contributes to cell survival, migration and neovascularization.

In conclusion, genetic engineering based bio-orthogonal non-canonical amino acid tagging is a powerful tool, enabling global-scale analysis of dynamic MSC proteomes *in vivo*. The method allows a better molecular understanding of MSC adaptive and reparative activities real time. Our results also suggest that MSC-derived DAMPs and complements may potentially be involved in the cardiac tissue repair.

## Supplementary Material

Supplementary figures and tables.

Supplementary table S1.
