# Supplementary material for: Analysis of mesenchymal stem cell proteomes in situ in the ischemic heart
Source: Theranostics. 2020 Sep 14;10(24):11324–38. doi: 10.7150/thno.47893 (PMC7532665; doi:10.7150/thno.47893)
Supplement: Supplementary file 1 — Supplementary figures and tables. [file thnov10p11324s1.pdf]

Analysis of mesenchymal stem cell proteomes in situ in the ischemic heart

Dunzheng Han[1,2,*], Junjie Yang[1,*], Eric Zhang[1], Yanwen Liu[1], Chan Boriboun[1], Aijun Qiao[1], Yang Yu[1], Jiacheng Sun[1], Shiyue Xu[1], Liu Yang[1], Wenying Yan[3], Bihui Luo[2], Dongfeng Lu[2], Chunxiang Zhang[1], Chunfa Jie[4], James Mobley[5], Jianyi Zhang[1], Gangjian Qin[1,#]

Supplementary Information

Supplementary Figures

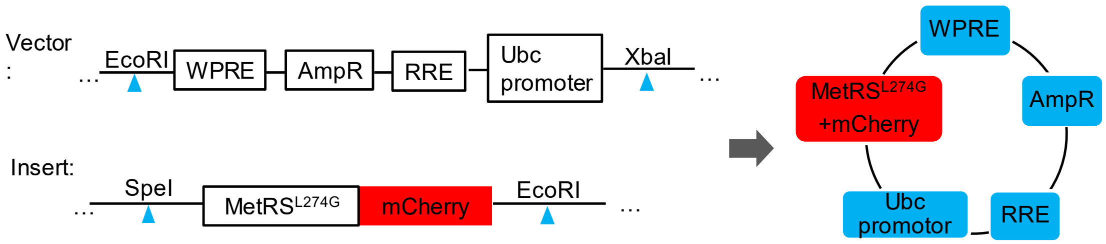

Figure S1: Cloning of lentiviral vector MetRS^{L274G}-mCherry: (*Upper left* panel) Backbone plasmid FUGW were digested with restriction enzyme EcoRI and XbaI to obtain a 9.1 kb fragment. (*Lower left* panel) MetRS^{L274G}-mCherry insert was created by amplifying a 3.6kb fragment of plasmid MaRSC via PCR with primers containing SpeI and EcoRI restriction sites at the ends (primer sequences reported in Methods), then digested with restriction enzyme SpeI and EcoRI (*Right* panel) The lentiviral plasmid MetRS^{L274G}-mCherry was obtained by ligating backbone and insert.

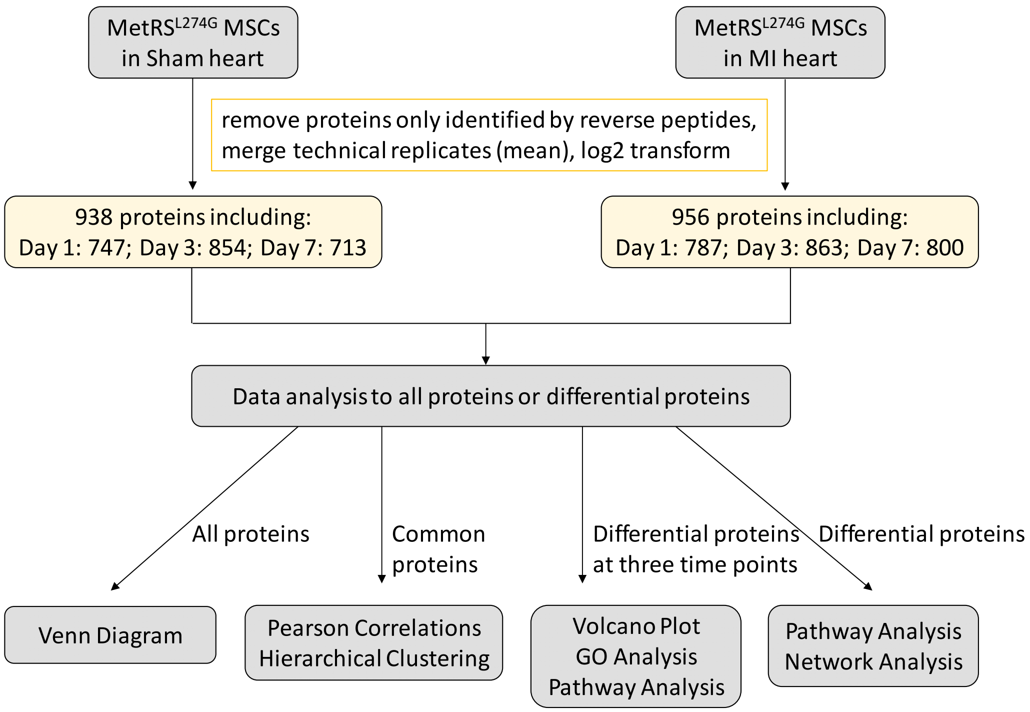

Figure S2. Workflow of mass spectrometry and data analysis.

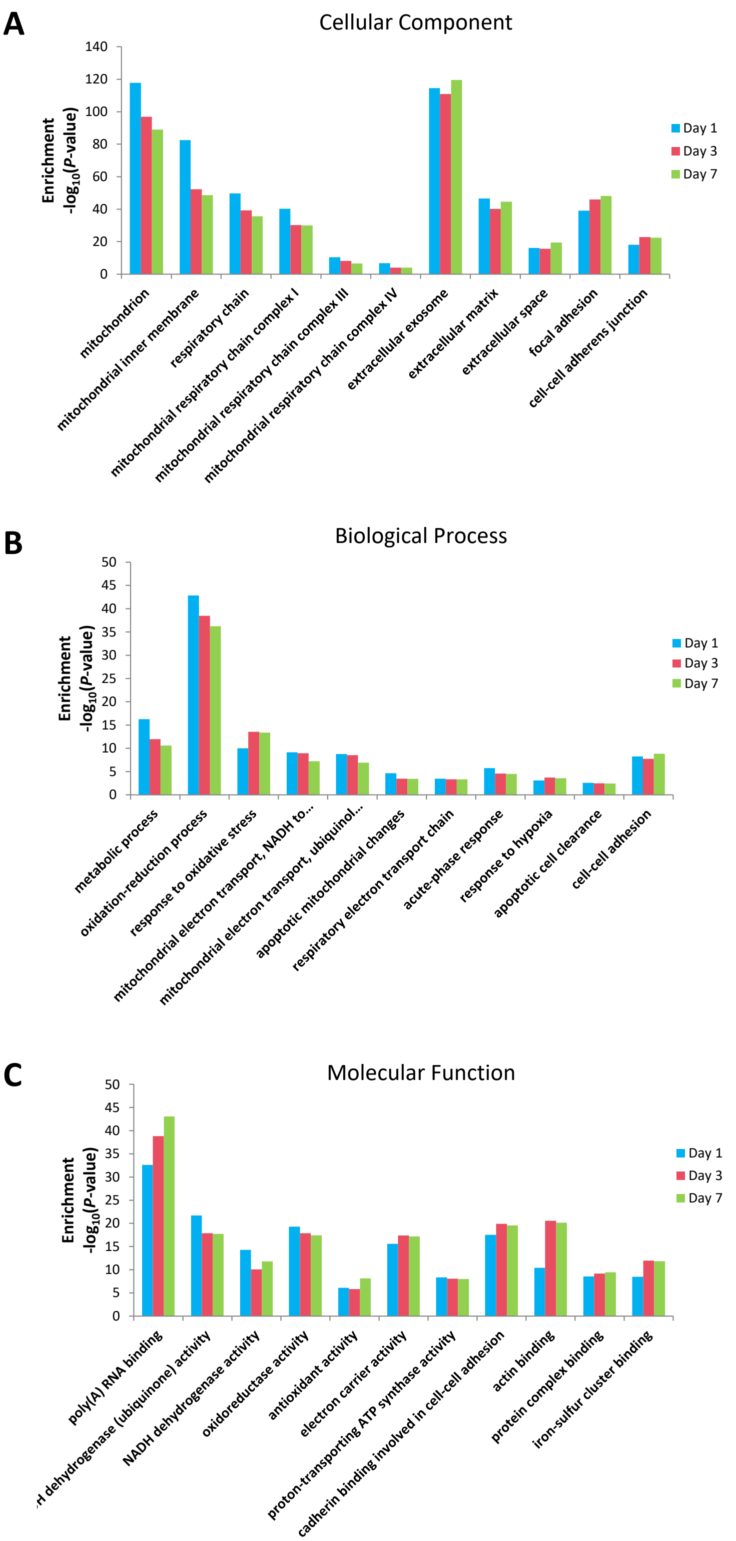

Figure S3: Gene Ontology analyses of MSC proteomes in the Sham heart: The significantly enriched GO terms for cellular components (**A**), biological processes (**B**) and molecular function (**C**) at day 1, 3, or day 7 in the Sham hearts. n=5 for each time point.

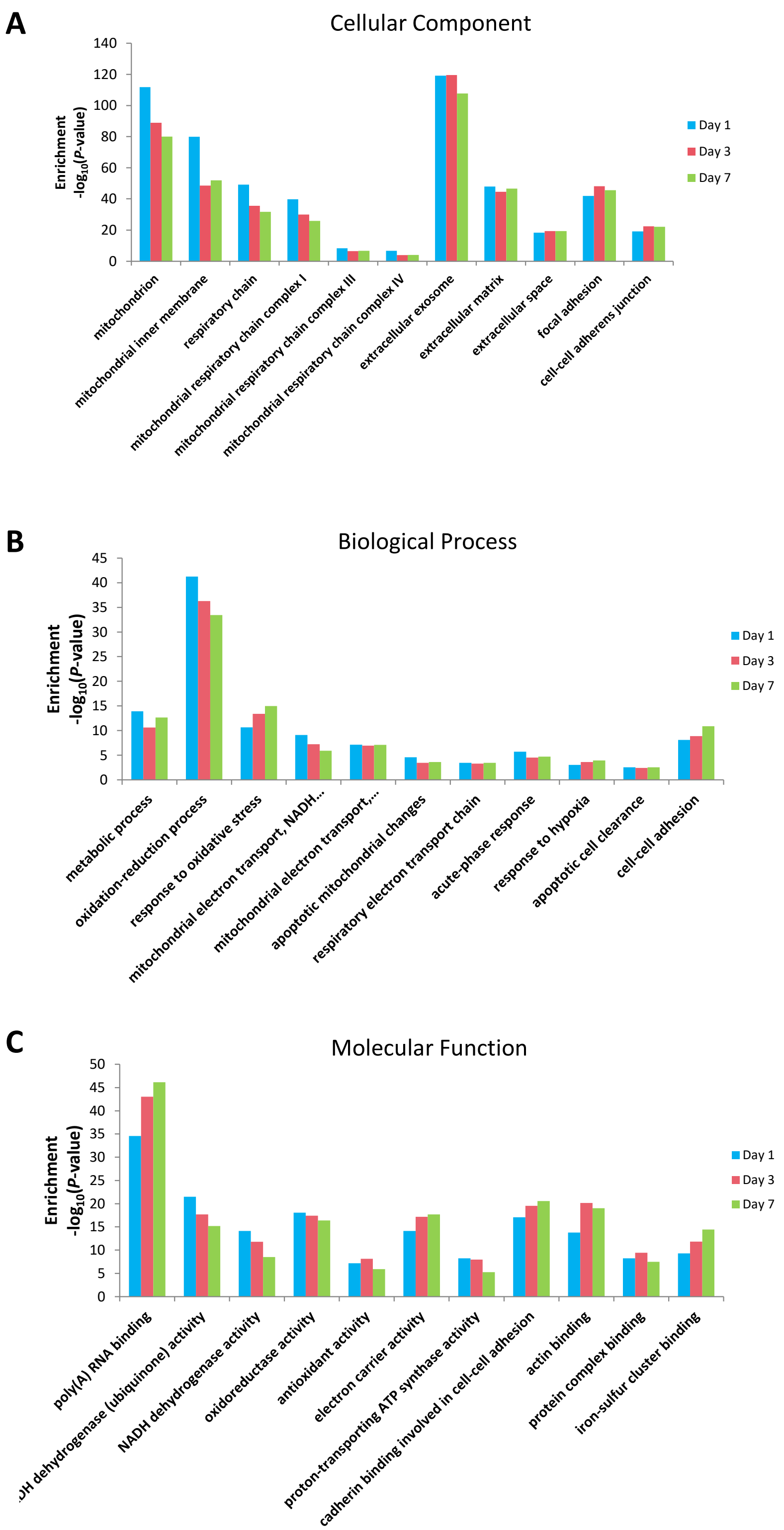

Figure S4: Gene Ontology analysis of MSC proteomes in the MI heart: The significantly enriched GO terms for cellular components (**A**), biological processes (**B**) and molecular function (**C**) at day 1, 3 or 7 in the MI hearts. n=5 for each time point.

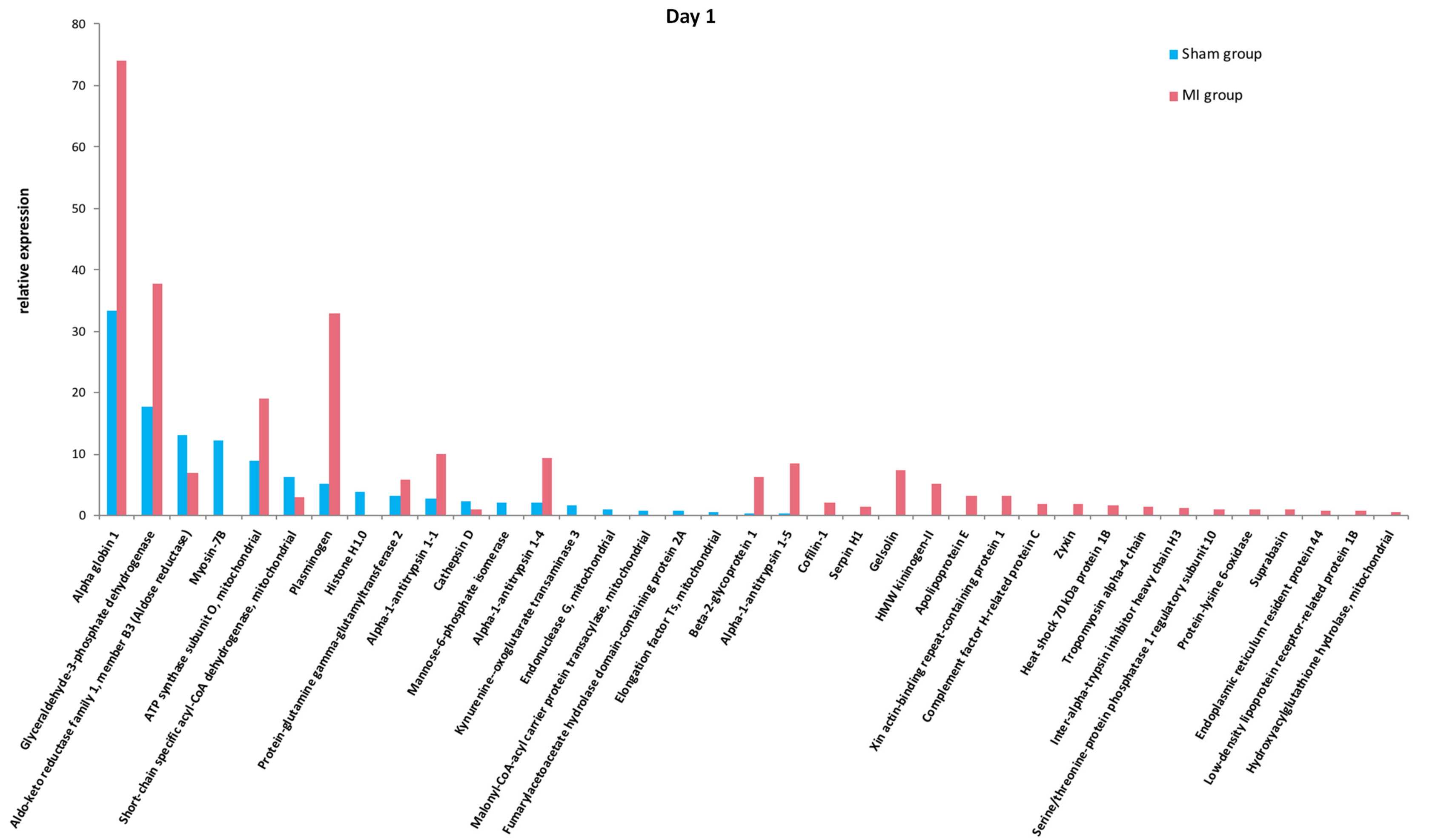

Figure S5: The relative expression levels of the differentially expressed proteins (fold change ≥1.5) in the MSCs transplanted for 1 day in the MI vs. Sham heart.

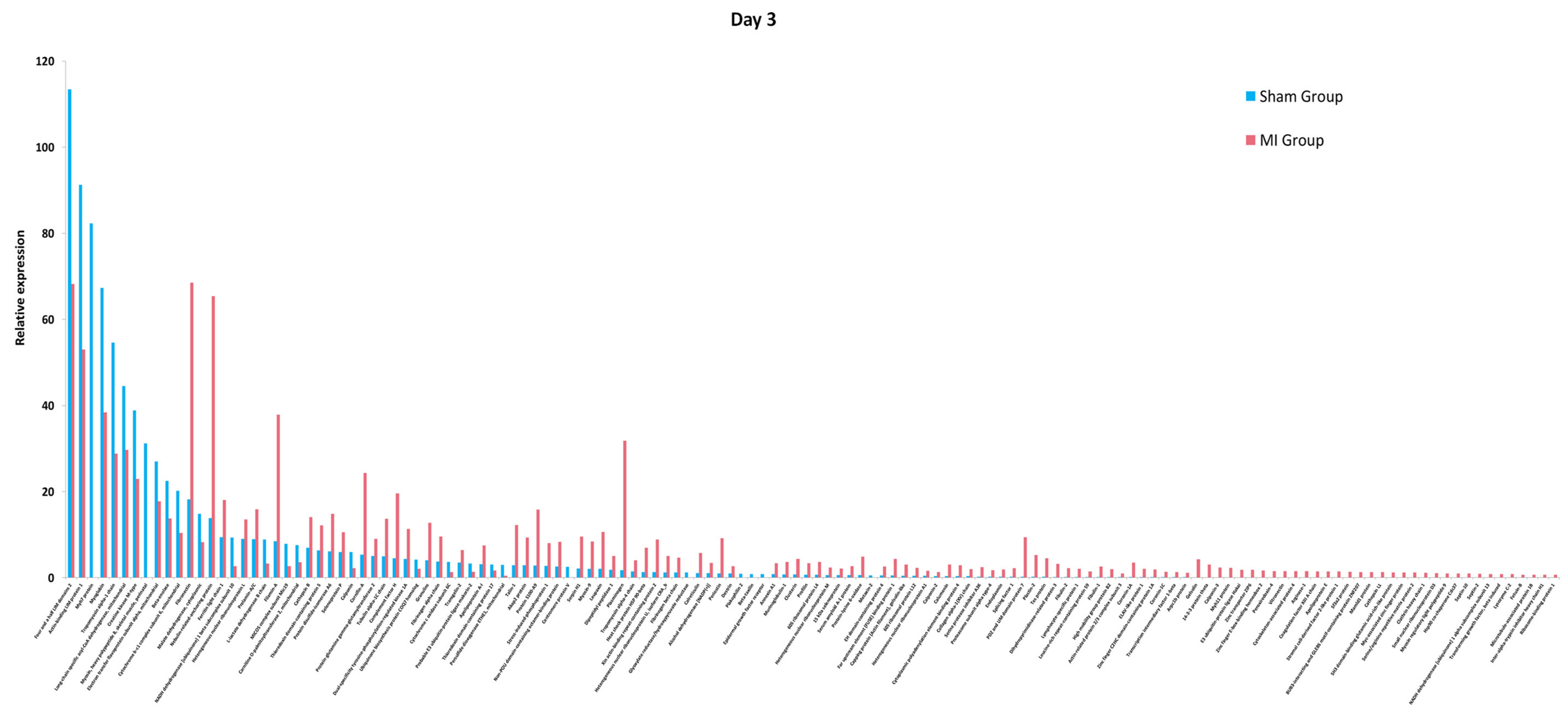

Figure S6: The relative expression levels of the differentially expressed proteins (fold change ≥1.5) in the MSCs transplanted for 3 days in the MI vs. Sham heart.

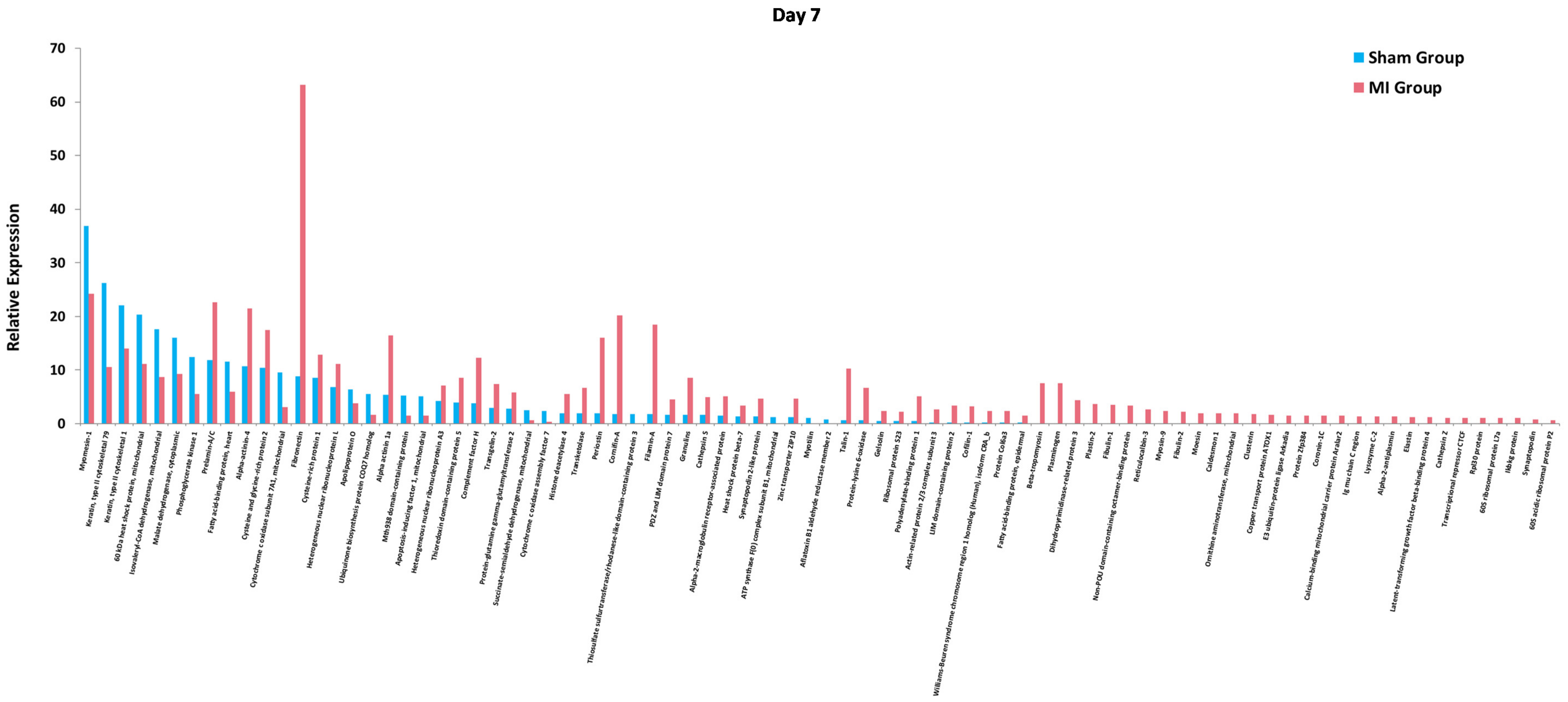

Figure S7: The relative expression levels of the differentially expressed proteins (fold change ≥1.5) in the MSCs transplanted for 7 days in the MI vs. Sham heart.
